# The validation and reliability of a Japanese version of the Problematic Online Gaming Questionnaire (POGQ-J)

**DOI:** 10.1186/s13722-021-00273-3

**Published:** 2021-11-20

**Authors:** Kazuya Inoue, Kengo Yokomitsu, Tomonari Irie, Makoto Matsuyama, Masanori Tanaka

**Affiliations:** 1 Faculty of Human Sciences, Waseda University, Tokorozawa, Saitama Japan; 2 Kawasaki University of Medical Welfare, Kurashiki, Okayama Japan; 3 School of Education and Culture, Hokusho University, Ebetsu, Hokkaido Japan; 4 College of Comprehensive Psychology, Ritsumeikan University, Ibaraki, Osaka Japan; 5 Department of Management, Faculty of Business Administration, Hokkai-Gakuen University, Sapporo, Hokkaido Japan

**Keywords:** Problematic Online Gaming Questionnaire (POGQ), Reliability, Validation, Japanese, Problematic online gaming

## Abstract

**Background:**

The Problematic Online Gaming Questionnaire (POGQ) is an 18-item self-rated measure designed to assess the degree of problematic online gaming. This study translated the POGQ into Japanese (POGQ-J) and examined the POGQ-J’s factor structure, validity, and reliability for a Japanese population.

**Method:**

A total of 285 undergraduate students (128 males, 157 females, *M*_age_ = 19.66, *SD* = 1.36) participated in this study.

**Results:**

A confirmatory factor analysis indicated the appropriateness of the POGQ-J’s six-factor structure, *χ*^2^(129) = 106.027, *p* < .931; *CFI* = .957; *RMSEA* = .040; *SRMR* = .054. Regarding convergent validity, the POGQ-J was found to be related to the time spent on online gaming (*r* = .309), the Game Addiction Scale for Adolescents (*r* = .824), and Young’s Internet Addiction Test (*r* = .581). Finally, the POGQ-J was found to have a high test–retest reliability.

**Conclusions:**

The POGQ-J is valid and reliable for assessing problematic online gaming in a Japanese population.

**Supplementary Information:**

The online version contains supplementary material available at 10.1186/s13722-021-00273-3.

## Background

The number of people engaged in online gaming has increased substantially with the spread of smartphones. Internet gaming disorder (IGD) is included in the fifth edition of the Diagnostic and Statistical Manual of Mental Disorders (DSM-5) [1], and the importance of treatment and research relating to IGD is increasing. According to a meta-analysis of 16 studies, the prevalence of IGD in adolescents was 4.6% (95% CI = 3.4–6.0%), but there is a clear gender imbalance among those diagnosed with IGD. Specifically, in males the prevalence was 6.8% (95% CI = 4.3–9.7%), while in females it was 1.3% (95% CI = 0.6–2.2% [2]). Moreover, IGD risk groups have a variety of comorbid mental health problems (e.g., somatization, obsession-compulsion, interpersonal sensitivity, depression, anxiety, hostility, phobic anxiety, paranoid ideation, and psychoticism) [3]. Given these problems associated with IGD, appropriate treatment and moderation in playing online games should be promoted to address the problem of excessive online gaming.

A Japanese study comprising a mail survey of 853 Japanese junior high school students in a suburban area (response rate: 97.6%; age: 12–15) using Young’s Internet Addiction Test: IAT (generally average: 20–39, moderate: 40–69, severe: 70–100) revealed that 2% scored 70 or higher and 21.7% scored between 40 and 69 [4]. In addition, a questionnaire survey of 5,096 randomly sampled Japanese 10–29-year-olds revealed that 85% (92.6% of men and 77.4% of women) had played games in the past year. Among them, 48.1% reported that they mainly played online games. When asked how much time was spent playing games on weekdays, 26.0% of men reported less than an hour per day, 30.4% reported one hour to less than two hours per day, 18.9% reported two hours to less than three hours per day, and 24.6% reported three hours or more per day. For females, 57.1% reported playing for less than an hour per day, 23.1% played one hour to less than 2 h, 9.3% played two hours to less than three hours, and 10.4% played for three hours or more per day [5]. Furthermore, factors that have been shown to increase the probability of Internet dependence include gaming and being under 30 years of age (odds ratio: 2.50 and 2.69, respectively) [6].

Few outpatient clinics in Japan specialize in online gaming-related health problems and are not able to provide sufficient support to those who need treatment. Thus, it is essential to accumulate evidence concerning basic knowledge for the treatment of problematic online gaming. Incidentally, there are several terms related to IGD, but in this paper, we will use the term “problematic online gaming,” based on the original study [7]. This term describes the quintessence of the phenomenon (i.e., the behavior is not only excessive, but there also is a presence of gaming-related problems), while avoiding the notion of dependency (as the exact definition and diagnostic criteria have not yet been clarified or agreed upon by researchers in this area). In Japan, such research on problematic online gaming is still in its nascent stage, and this topic has not been thoroughly explored. A reason for this is that a set of indicators for assessing symptoms related to problematic online gaming have not yet been developed. The Problematic Online Gaming Questionnaire (POGQ) devised by Demetrovics et al. [7] is a widely used tool for measuring the degree of problematic online gaming. The POGQ comprises 18 items with a six-factor structure. Using this scale, it is possible to understand individual and social problems caused by online games through six factors (preoccupation, overuse, immersion, social isolation, interpersonal conflict, and withdrawal). It is also possible to measure the problems faced by online game users from various angles. The POGQ is useful as a measurement tool and helps investigate clinical problems such as social isolation, interpersonal conflict, and withdrawal. The POGQ has been translated into Finnish [8], shortened [9], and used in a wide range of regions and cultures.

In Japan, there is a scale to measure the degree of game dependence, the Game Addiction Scale 7-Japanese version (GAS7-J) [10]; it has a one-factor structure. In contrast, the POGQ is a multifaceted scale that can assess related symptoms such as social isolation, interpersonal conflict, and withdrawal from online game dependence. Therefore, it is necessary to translate the POGQ and construct a Japanese version to address the problems that accompany online gaming in Japan. Doing so will allow us to research the effects of treatment for excessive online gaming of treatment on people. Translation of the POGQ to Japanese will allow us to develop a scale that can be used in clinical practice as an assessment indicator. Therefore, this study aims to translate the POGQ and verify its validity and reliability for assessing university students who play online games. We focused on university students because previous studies [7] have focused on young people (*M*_age_ = 21.01, *SD* = 5.85).

## Methods

### Participants

A total of 414 undergraduate students completed the questionnaires. The participants were recruited from Ritsumeikan University, Meisei University, Waseda University, Hokusyo University, and Hokkai-Gakuen University by distributing a flyer with a Google Forms survey link in the classrooms between December 1, 2019, and June 30, 2020. The participants from these five universities were taking a psychology course. They responded to the survey using their smartphones or PCs via the Google form URL on the flyer. The flyer stated the purpose of the study as “This survey is being conducted to determine daily life mood and thoughts about online games.” The flyer also contained the following statement: “The results of this study will be used for research purposes only. No individual will be identified. Your responses will not affect your academic performance. It will take approximately 10 min to complete.”

The target sample of this study was university students. One graduate student and one person over the age of 60 responded to the questionnaire and were excluded from the data analysis as they did not fit our criteria for inclusion. In addition, 127 respondents who answered that they did not play online games during the past week were excluded, resulting in 285 respondents (128 males, 157 females, *M*_age_ = 19.66, *SD* = 1.36) in the final analysis of the present study. This study was conducted in accordance with the COSMIN checklist [11], following the detailed guidelines of the preferred reporting style for the development of patient-reported outcome measures [12]. In this study, we did not use statistical methods to calculate the sample size. Mokkink et al. [13] state 7 times the number of items and ≥ 100 as a criterion of excellence as a guide for sample size when conducting factor analysis. Since the POGQ-J has 18 items, it was determined that a minimum of 126 participants would be needed. Since 285 subjects were included in the final analysis of this study, Mokkink’s [13] criteria were considered to be met. It was also necessary to gather over 100 people for the analysis criteria of test–retest reliability [13], and we collected as many samples as possible, considering the withdrawal rate of the second response for test–retest reliability of the POGQ-J. Moreover, no incentives were provided in this study.

### Measures

#### Demographics

Participants were asked questions to elicit information about their sex, age, and year of study in the university. Moreover, they were asked to report subjectively about the time and amount of money spent on online gaming in the previous week, the primary online games played, and overall Internet usage time in the previous week. The criterion of the past week was based on the original version of the paper [7]. All these items were answered via a Google form.

#### The Japanese version of the Problematic Online Gaming Questionnaire (POGQ-J; see Additional file 1)

The POGQ developed by Demetrovics et al. [7] comprises 18 items rated on a 5-point Likert scale (1 = *never*, 5 = *always*), with higher scores reflecting a greater tendency toward online problematic gaming. This scale consists of six factors (preoccupation: 2 items, overuse: 3 items, immersion: 4 items, social isolation: 3 items, interpersonal conflict: 2 items, and withdrawal: 4 items). The translation of the POGQ was conducted in accordance with the International Society for Pharmacoeconomics & Outcomes Research (ISPOR) task force [14]. First, a forward translation from English to Japanese was performed independently by two of the authors (KY and TI). Then, a professional English translator who was English-Japanese bilingual and blind to the original POGQ translated the provisional POGQ-J back into English. The two English versions of the POGQ (i.e., the original and back-translated versions) were reconciled by KY and TI, and only minor discrepancies were found. These discrepancies were discussed until a consensus was reached. The original author (DZ) evaluated the finalized English version of the POGQ-J and confirmed that the original meanings of each item, instructions, and responses were maintained throughout the translation procedure. As with the original POGQ, each item in the POGQ-J was rated by respondents on a 5-point Likert scale to indicate the extent to which they agreed with the values expressed in each item [7].

#### The Japanese version of the Game Addiction Scale for Adolescents (GAS7-J [10])

The GAS7-J comprises seven items rated on a 5-point Likert scale (1 = *never*, 5 = *very often*), with higher scores reflecting a greater tendency toward game addiction. These items include “Did you think about playing a game all day long?”; “Did you spend increasing amounts of time on games?”; “Did you play games to forget about real life?”; “Have others unsuccessfully tried to reduce your game use?”; “Have you felt bad when you were unable to play?”; “Did you have fights with others (e.g., family, friends) over your time spent on games?”; “Have you neglected other important activities (e.g., school, work, and sports) to play games?” The Japanese version of the GAS7-J has been shown to have good internal consistency (Cronbach’s α = .87) and a one-factor structure. The GAS7-J has been found to be correlated with game usage time (*r* = .32), the UCLA Loneliness Scale (*r* = .17), and the “Physical Aggression” subscale of the Japanese version of the Buss-Perry Aggression Questionnaire (*r* = .11). The GAS7-J was used in this study to examine the convergent validity of the POGQ-J. We assumed that the POGQ-J and GAS7-J would show a moderate positive correlation, as they are comprised of almost similar items (Items of POGQ-J: preoccupation, overuse, immersion, social isolation, interpersonal conflict, and withdrawal; items of GAS7-J: salience, tolerance, mood modification, relapse, withdrawal, conflict, and problems). Although there is a lot of overlap between the items, we expected that there would not be a high correlation because some factors are not common, such as preoccupation and social isolation.

#### The Japanese version of Young’s Internet Addiction Test (IAT) by the Kurihama Medical and Addiction Center (Treatment of Internet Addiction and Research: TIAR)

The IAT comprises 20 items rated on a 5-point Likert scale (1 = *rarely*, 5 = *always*), with higher scores reflecting a greater tendency toward Internet addiction. The IAT has been widely used in previous studies [4, 15]. We used the items translated by the Kurihama Medical and Addiction Center (TIAR) [16]. The IAT was used in this study to examine the discriminant validity of the POGQ-J. We assumed that the POGQ-J and IAT would show a moderate positive correlation, as people with tendencies of online game dependence have been shown to have higher IAT scores [17]. These items include “How often do you find that you stay online longer than you intended?” and “How often do you neglect household chores to spend more time online?”.

#### The Japanese version of the EuroQol 5 Dimension 5-level (EQ-5D-5L) [18]

This is a generic instrument used to assess the quality of life. It comprises five items on mobility, self-care, usual activity, pain/discomfort, and anxiety/depression. For each item, there are five possible levels of response, namely “no problems,” “slight problems,” “moderate problems,” “severe problems,” and “extreme problems,” with higher scores reflecting a greater tendency toward a poor health state. For mobility, the items are: “I have no problems in walking about,” “I have slight problems in walking about,” “I have moderate problems in walking about,” “I have severe problems in walking about,” and “I am unable to walk about.” For self-care, the items are: “I have no problems with washing or dressing myself,” “I have slight problems with washing or dressing myself,” “I have moderate problems with washing or dressing myself,” “I have severe problems with washing or dressing myself,” and “I am unable to wash or dress myself.” For daily or usual activities, the items are: “I have no problems doing my usual activities,” “I have slight problems doing my usual activities,” “I have moderate problems doing my usual activities,” “I have severe problems doing my usual activities,” and “I am unable to do my usual activities.” For pain/discomfort, the items are: “I have no pain or discomfort,” “I have slight pain or discomfort,” “I have moderate pain or discomfort,” “I have severe pain or discomfort,” and “I have extreme pain or discomfort.” The items for anxiety/depression are: “I am not anxious or depressed,” “I am slightly anxious or depressed,” “I am moderately anxious or depressed,” “I am severely anxious or depressed,” and “I am extremely anxious or depressed.”

The EQ-5D-5L was used in this study to examine the convergent validity of the POGQ-J. We assumed that the POGQ-J and EQ-5D-5L would show a moderate positive correlation as game dependence is associated with a variety of psychiatric problems [3], and among people with these problems, it is assumed that the quality of life is lower.

### Procedure

Participants were considered to have provided written informed consent for the use of the information provided for research purposes by completing the research questionnaire using Google Forms. To verify the test–retest reliability of the POGQ-J, participants who responded were asked to retake the POGQ-J approximately two weeks after their initial response.

### Statistical analysis

The analysis of the descriptive statistics, convergent and divergent validity, and intraclass correlation coefficients (ICCs) were conducted using IBM SPSS statistics software version 26. For the ICC criteria, .40–.59 was considered fair, .60–.74 good, and over .75 excellent [19]. A confirmatory factor analysis was carried out to confirm the factor structure of the POGQ-J. We hypothesized that the POGQ had a six-factor model. Because the POGQ-J items are rated on a 5-point Likert scale (an ordinal and categorical scale), a weighted least squares mean-variance (WLSMV) estimation was conducted. Four fit indices were employed: chi-square (χ^2^), comparative fit index (CFI), root mean square error of approximation (RMSEA), and standardized root mean square residual (SRMR). Confirmatory factor analysis was conducted using the Lavaan package with the statistical software package R 3.5.2 (R Core Team, 2019, Vienna, Austria). The goodness of fit criteria were set as *CFI* > .95, *RMSEA* < .05, and *SRMR* < .05 [20]. In this study, a p-value of less than 5% was considered significant.

## Results

### Demographic data

Table 1 summarizes the demographic characteristics of the participants.

**Table 1:** Demographics and gaming characteristics (*n* = 285)

Category	*n*	%
Sex		
Male	157	44.9
Female	128	55.1
Age		
17–18	78	27.4
19–20	120	42.1
More than 21	87	30.5
College year level		
First year	118	41.4
Second year	46	16.1
Third year	84	29.5
Fourth year	37	13.0
Time spent on online gaming (hours per week)		
0–1.5	50	17.5
2.0–4.5	65	22.8
5.0–12.0	89	31.2
More than 14	81	28.4
Time spent on the Internet (hours per week)		
0–9.00	68	23.9
10.00–25.62	71	40.7
28.00–48.00	57	20.0
More than 50.00	89	31.2
Money spent on online gaming (per month)		
None	236	82.8
10–980 (JPY^a^)	11	3.9
1000–7000 (JPY^a^)	23	8.1
10,000–50,000 (JPY^a^)	15	5.3

^a^Japanese Yen

### Descriptive statistics

Table 2 presents the means and standard deviations of each variable.

**Table 2:** Means, standard deviations, and internal consistencies (*n* = 285)

Variable	*M*	*SD*^e^	*α*^f^	Min	Lower quartile	Median	Upper quartile	Max
POGQ-J^a^	35.28	12.17	0.91	18	25.00	34.00	43.00	71
Preoccupation	3.79	1.71	0.56	2	2.00	4.00	5.00	10
Immersion	9.72	3.81	0.77	4	7.00	9.00	13.00	20
Withdrawal	6.47	3.16	0.83	4	4.00	5.00	8.00	19
Overuse	6.83	3.01	0.74	3	4.00	6.00	9.00	15
Interpersonal conflicts	3.00	1.54	0.72	2	2.00	2.00	4.00	10
Social isolation	5.46	2.43	0.64	3	3.00	5.00	7.00	15
GAS7-J^b^	11.98	4.70	0.79	7	8.00	11.00	14.00	30
IAT^c^	48.09	15.63	0.91	20	36.00	48.00	60.00	91
EQ-5D-5L^d^	5.84	1.29	0.55	5	5.00	5.00	6.00	15
Time spent on online gaming (hours)	11.03	13.52	–	0.05	2.00	5.00	14.50	70
Internet usage time (hours)	683.00	47.75	–	1	10.00	28.00	50.00	683
Money spent on online gaming (JPY^g^)	1292.8	5181.69	–	0	0.00	0.00	0.00	5000

^a^Japanese version of the Problematic Online Gaming Questionnaire

^b^Japanese version of the Game Addiction Scale for Adolescents

^c^Young’s Internet Addiction Test

^d^EuroQol 5 Dimension 5-level

^e^Standard deviation

^f^Cronbach’s alpha

^g^Japanese Yen

### Confirmatory factor analysis of the POGQ-J

**Fig. 1:**
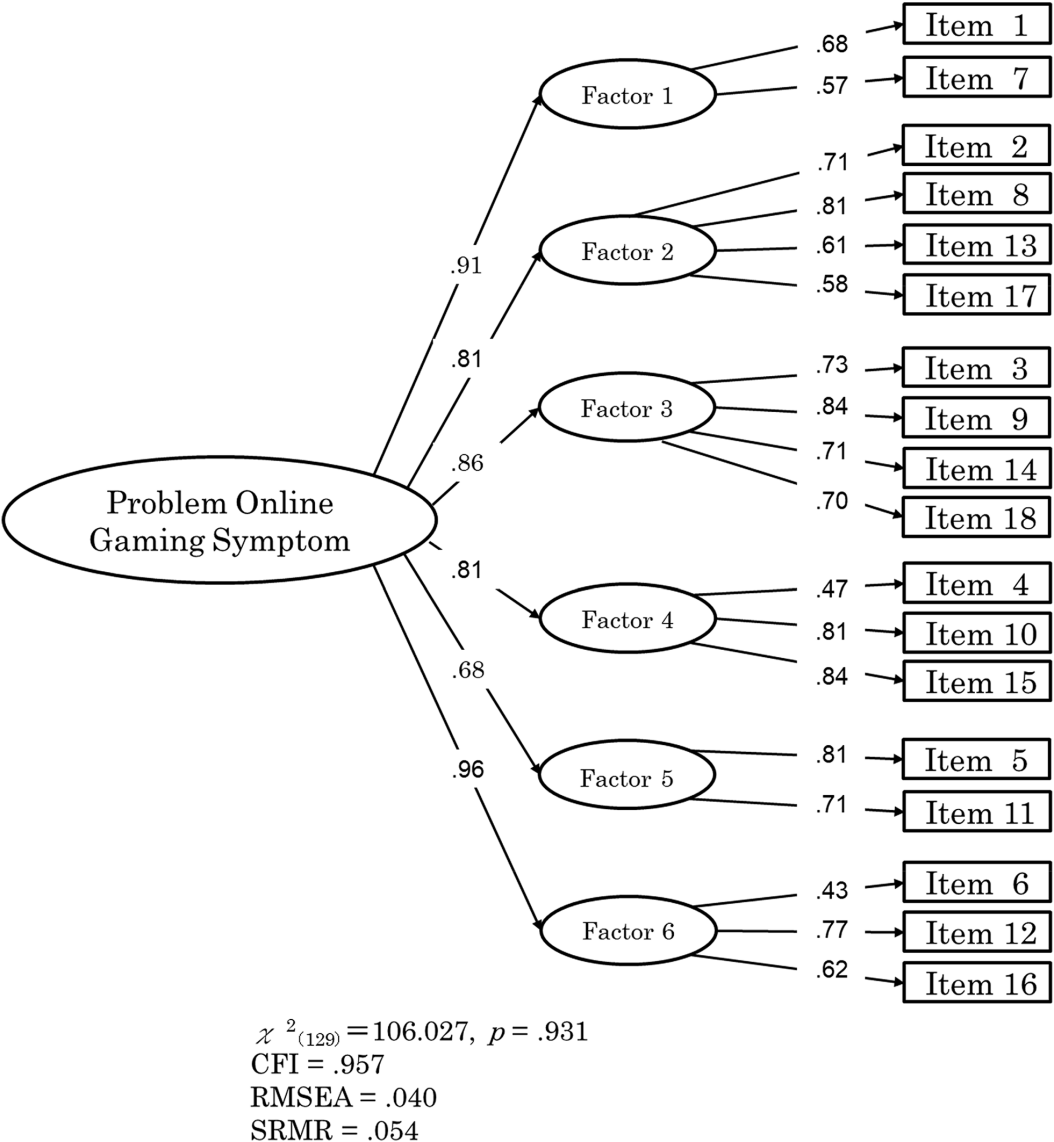
Confirmatory factor analysis model of the POGQ-J. Factor structure of the Japanese version of the Problem Online Gaming Questionnaire. Note 1. Factor 1 = Preoccupation; Factor 2 = Immersion; Factor 3 = Withdrawal; Factor 4 = Overuse; Factor 5 = Interpersonal Conflicts; Factor 6 = Social Isolation. Note 2. *CFI *comparative fit index, *RMSEA* root mean square error of approximation, *SRMR* standardized root mean square residual

To assess the similarity of the POGQ-J factor structure with that of the original POGQ, we conducted a confirmatory factor analysis using a weighted least squares mean–variance (WLSMV) estimation to test the six-factor structure of the POGQ-J. The results of this analysis showed that the fit indices used were acceptable (Fig. 1). The POGQ-J was found to exhibit a six-factor structure similar to that of the original POGQ, confirming the goodness of fit of the tolerance range.

### Internal consistency of the POGQ-J

The Cronbach’s alpha for POGQ-J is shown in Table 2.

### Convergent validity of the POGQ-J

Regarding convergent validity, the POGQ-J was related to the time spent on online gaming (*r* = .309, *p* < .001), the Game Addiction Scale for adolescents (*r* = .824, *p* < .001), and the EQ-5D-5L (*r* = .291, *p* < .001; Table 3).

**Table 3:** Convergent and discriminant validity for POGQ-J

Variable	POGQ-J	GAS7-J	IAT	EQ-5D-5L
POGQ-J^a^				
GAS7-J^b^	.824***			
IAT^c^	.581***	.582***		
EQ-5D-5L^d^	.291***	.353***	.350***	
Time spent on online gaming	.309***	.327***	.120*	.070

*p < .05, **p < .01, ***p < .001

^a^Japanese version of the Problematic Online Gaming Questionnaire

^b^Japanese version of the Game Addiction Scale for Adolescents

^c^Young’s Internet Addiction Test

^d^EuroQol 5 Dimension 5-level

As a supplementary analysis, we calculated the correlation coefficients for each factor of EQ-5D-5L and POGQ-J. The POGQ-J showed a weak correlation between usual activity and anxiety/depression (Table 4).

**Table 4:** Correlation between POGQ-J and each factor of the EQ-5D-5L

Variable	Mobility	Self-care	Usual activity	Pain/ Discomfort	Anxiety/ Depression
Mobility					
Self-care	.331***				
Usual activity	.328***	.414***			
Pain/Discomfort	.394***	.303***	.302***		
Anxiety/Depression	.180**	.081	.311***	.385***	
POGQ-J	.136*	.079	.290***	.121*	.245***

*p < .05, **p < .01, ***p < .001

### Discriminant validity of the POGQ-J

Regarding discriminant validity, POGQ-J showed a moderate correlation with Young’s IAT (Table 3).

### Test–retest reliability of the POGQ-J

The *ICC* of the POGQ-J scores was analyzed at two-time points. A total of 73 participants (35 male, 38 female, *M*_age_ = 19.74, *SD* = 1.29) were included in the analysis. The test–retest reliability of the POGQ-J fulfilled the criteria for being excellent, with *ICC* (2,1) = .838, *p* < .001, and 95% CI = .754–.895.

## Discussion

This study translated the POGQ into Japanese (POGQ-J) and examined the translated scale’s factor structure, validity, and reliability for a young Japanese population. The POGQ-J was confirmed to have a six-factor structure. Additionally, the convergent validity of the POGQ-J was demonstrated by its association with time spent on online gaming and the Game Addiction Scale for Adolescents. In addition, the discriminant validity of the POGQ-J was confirmed by its association with Young’s IAT. Moreover, the POGQ-J showed high test–retest reliability. The results show that the POGQ-J is a valid and reliable scale that can measure the degree of online game dependence on six dimensions.

The six-factor structure of the POGQ-J was shown to be consistent with that of the original POGQ [7]. The POGQ-J was also confirmed to have a high internal consistency (Cronbach’s α = .91), similar to that of the original POGQ (Cronbach’s α = .93). Thus, the results of this study support previous findings that show that the POGQ has a six-factor and sufficient internal consistency.

Regarding the scale’s convergent validity, the POGQ-J showed a moderate positive correlation with regard to time spent on online gaming. This result was considered reasonable. In addition, the POGQ-J showed a high positive correlation with the GAS7-J. The correlation coefficients were higher than we hypothesized. These results suggest that the POGQ-J is an instrument that focuses on problematic online gaming and is hence a valuable indicator in this research area.

The POGQ-J showed a weak positive correlation with the EQ-5D-5L. A supplementary analysis revealed a significant weak positive correlation between the total score of the POGQ-J and the scores of usual activity and anxiety/depression items of the EQ-5D-5L. The usual activity score represents the degrees of work, study, family, and leisure activities. Although the causal relationship is unclear, the results suggest that there is an association between high POGQ-J scores and the degree to which usual activities are inhibited. A significant positive weak correlation between the POGQ-J total score and the anxiety/depression score of the EQ-5D-5L was also confirmed. This result was mostly consistent with a previous study [3]. Therefore, correlations between these scores indicate that the POGQ-J has good convergent validity. The correlations between POGQ-J and EQ-5D-5L were lower than expected, likely because this study was conducted during a period in which an increased number of people were staying at home due to COVID-19.

As for the discriminant validity of the POGQ-J, the POGQ-J showed a moderate positive correlation with Young’s IAT. This result generally supports the hypothesis of this study and confirms the discriminant validity of the POGQ-J. However, the POGQ-J and the IAT showed a higher positive correlation than we hypothesized. Presumably, the POGQ-J’s focus on online gaming led to a higher correlation with the IAT, which measures Internet addiction. Lastly, regarding the test–retest reliability of the POGQ-J that fulfilled the criteria as excellent [19].

### Limitations

Even though this study included students of different ages, gender, faculties, and universities, there may still be sampling biases; this is important to consider when interpreting the results of this online study. Moreover, a potential limitation of this study was its offline and non-clinical sampling method. Although the psychometric properties of the POGQ-J were good, it is not clear whether the results of this study can be generalized outside an offline sample, such as in an online or clinical sample. As the POGQ-J is expected to be used in both a non-clinical population as well as in the clinical assessment and treatment of IGD, it is necessary to assess the psychometric properties of the POGQ-J and to replicate the factor structure, reliability, and validity with online and clinical samples. In addition, The POGQ-J fails to assess the symptoms of tolerance, continued use, deception, and escape in the internet gaming disorder section of the DSM-5 [21]. Therefore, it is also important to conduct more extensive research on game-related symptoms. For example, it would be helpful to use indicators such as the Internet gaming disorder scale–short-form (IGDS9-SF) [22].

## Conclusion

A Japanese version of the POGQ was constructed, and its reliability and validity were confirmed. The POGQ-J was confirmed to have a six-factor structure, similar to that of the original POGQ. Moreover, the convergent validity and the discriminant validity of the POGQ-J were shown, and the POGQ-J showed a high test–retest reliability. The POGQ-J covers multiple types of factors, such as preoccupation, overuse, immersion, social isolation, interpersonal conflict, and withdrawal. The advantage of the POGQ-J scale is that it makes it possible to measure, from different dimensions, the different aspects that depend on online games. Through the POGQ-J, it becomes possible to understand game addiction trends and the interpersonal problems, social isolation, and degree of withdrawal caused by game addiction. The use of the POGQ-J is expected to further promote research on online game-dependence treatment and prevention.

## Supplementary Information

**Additional file 1.** The Problematic Online Gaming Questionnaire-Japanese version (POGQ-J).

## Data Availability

The datasets generated and/or analyzed during the current study are available in the Open Science Framework: OSF repository, https://osf.io/jvw5f/?view_only=90b04607e7b344c08aa005068ea34f84.

## Abbreviations

CFI: Comparative fit index
EQ-5D-5L: EuroQol 5 Dimension 5-level
ICC: Intraclass correlation coefficients
IGD: Internet gaming disorder
ISPOR: International Society for Pharmacoeconomics & Outcomes Research
POGQ: Problematic Online Gaming Questionnaire
RMSEA: Root mean square error of approximation
SRMR: Standardized root mean square residual
WLSMV: Weighted least squares mean–variance
