# Supplementary material for: The validation and reliability of a Japanese version of the Problematic Online Gaming Questionnaire (POGQ-J)
Source: Addict Sci Clin Pract. 2021 Nov 20;16:69. doi: 10.1186/s13722-021-00273-3 (PMC8605469; doi:10.1186/s13722-021-00273-3)
Supplement: Supplementary file 1 — Additional file 1. The Problematic Online Gaming Questionnaire-Japanese version (POGQ-J). [file 13722_2021_273_MOESM1_ESM.docx]

**Additional file 1.** The Problematic Online Gaming Questionnaire-Japanese version (POGQ-J)

| オンラインゲームに関する以下の項目を読んで、自分にどの程度あてはまるか、１から５の数字で回答してください。なお、このアンケートは、「オンラインゲームに関するアンケート」ですが、それぞれの質問項目では「ゲーム」と簡単に表記しています。 | Never  全くない | Seldom  めったにない | Occasionally  たまにある | Often  よくある | Always  ほとんどいつも |
| --- | --- | --- | --- | --- | --- |
| 1. When you are not gaming, how often do you think about playing a game or think about how it would feel to play at that moment?  ゲームをしていない時に、ゲームをプレイしている時のこと、あるいは今プレイするとどんな感情になるかについて、どのくらいの頻度で考えますか | **1** | **2** | **3** | **4** | **5** |
| 2. How often do you play longer than originally planned?  思っていた以上に長くゲームをプレイすることが、どのくらいの頻度でありますか | **1** | **2** | **3** | **4** | **5** |
| 3. How often do you feel depressed or irritable when not gaming only for these feelings to disappear when you start playing?  ゲームをしていない時に、ゲームを始めないと消えない落ち込みやイライラなどの感情を、どのくらいの頻度で感じますか | **1** | **2** | **3** | **4** | **5** |
| 4. How often do you feel that you should reduce the amount of time you spend gaming?  ゲームに費やしている時間を減らすべきであると、どのくらいの頻度で感じますか | **1** | **2** | **3** | **4** | **5** |
| 5. How often do the people around you complain that you are gaming too much?  周りの人達は、あなたがゲームをし過ぎていることに対して、どのくらいの頻度で不満や文句を言っていますか | **1** | **2** | **3** | **4** | **5** |
| 6. How often do you fail to meet up with a friend because you were gaming?  ゲームをしていたことが原因で、友人と会うことができなかったことが、どのくらいの頻度でありますか | **1** | **2** | **3** | **4** | **5** |
| 7. How often do you daydream about gaming?  ゲームをしている空想にふけることが、どのくらいの頻度でありますか | **1** | **2** | **3** | **4** | **5** |
| 8. How often do you lose track of time when gaming?  時がたつのを忘れるほどゲームをすることが、どのくらいの頻度でありますか | **1** | **2** | **3** | **4** | **5** |
| 9. How often do you get irritable, restless or anxious when you cannot play games as much as you want?  好きなだけゲームをプレイできない時に、イライラしたり、落ち着かなくなったり、不安になったりすることが、どのくらいの頻度でありますか | **1** | **2** | **3** | **4** | **5** |
| 10. How often do you unsuccessfully try to reduce the time you spend on gaming?  ゲームに費やしている時間を減らそうと努力して、うまく行かなかったことが、どのくらいの頻度でありますか | **1** | **2** | **3** | **4** | **5** |
| 11. How often do you argue with your parents and/or your partner because of gaming?  ゲームが原因で、親、配偶者、恋人などと口論することが、どのくらいの頻度でありますか | **1** | **2** | **3** | **4** | **5** |
| 12. How often do you neglect other activities because you would rather game?  ゲームのために、ほかの活動をないがしろにするようなことが、どのくらいの頻度でありますか | **1** | **2** | **3** | **4** | **5** |
| 13. How often do you feel time stops while gaming?  ゲームをしている最中に、時間が止まっていると感じることが、どのくらいの頻度でありますか | **1** | **2** | **3** | **4** | **5** |
| 14. How often do you get restless or irritable if you are unable to play games for a few days?  もし数日間ゲームをプレイできないような時に、そわそわしたり、いらいらしたりすることが、どのくらいの頻度でありますか | **1** | **2** | **3** | **4** | **5** |
| 15. How often do you feel that gaming causes problems for you in your life?  ゲームをすることによって、あなたの生活に問題が引き起こされると感じることは、どのくらいの頻度でありますか | **1** | **2** | **3** | **4** | **5** |
| 16. How often do you choose gaming over going out with someone?  誰かと外出するよりもゲームをすることを選ぶことが、どのくらいの頻度でありますか | **1** | **2** | **3** | **4** | **5** |
| 17. How often are you so immersed in gaming that you forget to eat?  ゲームに夢中になっているあまり、食べることを忘れることがどのくらいの頻度でありますか | **1** | **2** | **3** | **4** | **5** |
| 18. How often do you get irritable or upset when you cannot play?  ゲームができない時に、イライラしたり混乱したりすることが、どのくらいの頻度でありますか | **1** | **2** | **3** | **4** | **5** |

| preoccupation | immersion | withdrawal | overuse | interpersonal conflicts | social isolation |
| --- | --- | --- | --- | --- | --- |
| 1, 7 | 2, 8, 13, 17 | 3, 9, 14, 18 | 4, 10, 15 | 5, 11 | 6, 12, 16 |
